# The Entomopathogenic Fungus *Conidiobolus coronatus* Has Similar Effects on the Cuticular Free Fatty Acid Profile of Sensitive and Resistant Insects

**DOI:** 10.3390/insects14110895

**Published:** 2023-11-20

**Authors:** Mieczysława Irena Boguś, Michalina Kazek, Mikołaj Drozdowski, Agata Kaczmarek, Anna Katarzyna Wrońska

**Affiliations:** 1 Museum and Institute of Zoology, Polish Academy of Sciences, ul. Wilcza 64, 00-679 Warszawa, Poland; mikolajdrozdowski@gmail.com (M.D.); akaczmarek@miiz.waw.pl (A.K.);; 2 Department of Microbiology, Molecular Genetics and Genomics, Centre of Advanced Materials and Technology CEZAMAT, Warsaw University of Technology, ul. Poleczki 19, 02-822 Warsaw, Poland; michalina.kazek@pw.edu.pl

**Keywords:** *Galleria mellonella*, *Calliphora vicina*, *Conidiobolus coronatus*, cuticle, conidia, free fatty acids, GC/MS

## Abstract

**Simple Summary:**

To understand the mechanisms underlying the recognition of susceptible hosts by entomopathogenic fungi, it is critical to decipher the role of their cuticular lipids. The present study compared the effect of infection by the entomopathogenic fungus *Conidiobolus coronatus* (Constantin) Batko (Entomophthorales) on the larvae and adults of susceptible *Galleria mellonella* Linnaeus (Lepidoptera: Pyralidae) and resistant *Calliphora vicina* Robineau-Desvoidy (Diptera: Calliphoridae). The results indicate variations in the free fatty acid (FFA) profiles of the cuticles with regard to species and developmental stage and the changes occurring in them following contact with the fungus.

**Abstract:**

The mechanisms underlying the recognition of a susceptible host by a fungus and the role of cuticular compounds (CCs) in this process remain unclear; however, accumulated data suggest that this is influenced to a great degree by cuticular lipids. Two insect species differing in their sensitivity to fungal infection, viz. the highly sensitive *Galleria mellonella* Linnaeus (Lepidoptera: Pyralidae) and the resistant *Calliphora vicina* Robineau-Desvoidy (Diptera: Calliphoridae), exhibited significant qualitative and quantitative changes in cuticular free fatty acid (FFA) profiles after exposure to *Conidiobolus coronatus* (Constantin) Batko (Entomopthorales). Despite being systematically distant, leading different lifestyles in different habitats, both insect species demonstrated similar changes in the same FFAs following exposure to the fungus (C12:0, C13:0, C14:0, C15:0, C16:1, C16:0, C18:1, C18:0), suggesting that these are involved in a contact-induced defense response. As it was not possible to distinguish the share of FFAs present in the conidia that were attached to the cuticle from the FFAs of the cuticle itself in the total number of extracted FFAs, further research is necessary.

## 1. Introduction

A number of studies have shown that entomopathogenic fungi play a vital role as biological agents for controlling insect populations in the natural environment, and several species of entomopathogenic fungi have already been harnessed for this purpose. Many bioinsecticides based on fungi are in regular use in integrated pest management programs, offering environmentally friendly alternatives to synthetic chemical insecticides [1,2,3,4]. One of the most promising species of entomopathogens is *Conidiobolus coronatus*, a cosmopolitan soil fungus of the Entomophthorales, which is known to infect a number of insects and various collembolans [5,6] and is known to produce secondary metabolites harmful to insects [5,7,8,9,10,11,12]. After *C. coronatus* infection, non-resistant insects are quickly killed following organ and tissue damage caused by the growing hyphae [13,14,15], as well as by the release of toxic metabolites, which appear to play a key role in affecting the insect immune system [7,9,10,11,12,16,17,18,19].

Unlike entomopathogenic viruses and bacteria, which must enter the gastrointestinal tract of the host with food to cause infection, entomopathogenic fungi are armed with a set of proteolytic, chitinolytic and lipolytic enzymes that degrade the protein, chitin and lipid components of the insect cuticle, thus enabling direct penetration through the exoskeleton and access to the nutrient-rich hemocoel [20,21,22,23]. Research suggests that the specific structure and chemical composition of the exoskeleton play important parts in determining the sensitivity or resistance of various insect species to fungal infection; data indicate that conidial adherence and germination are strongly influenced by these cuticular components [15,24,25,26,27,28,29,30,31,32,33,34,35,36,37,38,39,40,41,42,43]. Cuticular compounds (CCs) have been found to strongly influence *C. coronatus* growth, sporulation and virulence, as well as the activity of cuticle-degrading enzymes (CDEs) when added to fungal cultivation medium [44,45,46,47]. Several CCs possess antibacterial and antifungal properties [48,49,50,51,52,53,54,55,56,57,58,59], while others stimulate the germination of fungal spores and hyphal growth and enhance virulence [45,46,60,61,62].

Many insect species possess an exoskeleton that provides an effective physical and chemical barrier to infection, which may reduce the costly immune defense inside the hemocoel [15,20,24,63]. Preventing penetration by investing in a well-equipped cuticle, thus improving the first line of defense against fungal invasive structures, is a much more effective strategy than attempting to repel an intruder that has breached a thinner and poorly defended cuticle; such investment is believed to be one of the factors behind the evolutionary success of scavenger flies and cockroaches [47,63].

*Calliphora vicina* larvae possess a thick cuticle that provides a very effective barrier against *C. coronatus*, and is it poorly degraded *in vitro* by proteases; the protective role of the cuticle is supported by the hemolymph, which has also demonstrated effective antiproteolytic capacity [15,47,63]. However, the immune response of *C. vicina* is generally weak, characterized by hemocytes with low phagocytic and encapsulating activity, an inefficient polyphenol oxidase (PO) system and a hemolymph with low lysozyme activity [15]. Therefore, an effective cuticle is essential for protecting *C. vicina* larvae, which, given their lifestyle and habitat, are constantly exposed to pathogens [15,64,65]. In contrast, *Galleria mellonella* larvae, despite having both humoral and cellular components in their immune systems, are vulnerable to *C. coronatus* infection because of their relatively thin and easily degraded cuticle [15,46].

The mechanisms underlying the fungal recognition of a susceptible host and the role of CCs in this process are still unclear; however, accumulated data suggest that a key role may be played by cuticular lipids [24,45,54,63]. This paper compares the changes that occur in the free fatty acid (FFA) profiles of *C. vicina* and *G. mellonella* cuticles after exposure to *C. coronatus*. The construction of these FFA profiles is specific to the species, sex, developmental stage and physiological state of the insect [66,67]. The main aim of the present work was to determine whether contact with *C. coronatus* triggers similar physiological processes in susceptible and resistant insect hosts following infection, manifested by changes in the FFA profiles of the cuticle.

## 2. Materials and Methods

### 2.1. Insects

Two species of insects differing in their sensitivity to infection with *C. coronatus* were compared: the highly sensitive *G. mellonella* (Lepidoptera: Pyralidae) and the resistant *C. vicina* (Diptera: Calliphoridae). The insects were reared in the laboratory under optimal growth conditions.

*Galleria mellonella* was reared in glass jars covered with cotton cloth at 30 °C, 70% relative humidity and in constant darkness on a semi-artificial diet prepared according to Sehnal [68]. The experiments were performed on five-day-old last (seventh) instar larvae, which had ceased feeding and entered the wandering stage, and on mature six-day-old adults.

*Calliphora vicina* was grown as described previously [63] at 25 °C, long day (L:D 16:8) and 70% relative humidity. Adult flies were kept in a glass container covered with miller’s gauze and had unlimited access to bovine meat and water supplemented with glucose. The larvae were fed fresh beef *ad libitum*. The insects were bred in the above conditions for six generations before starting the experiments. Larvae in the last (third) larval instar (wandering stage) and six-day-old mature adults were used in the experiments.

### 2.2. Fungus

*Conidiobolus coronatus* (Entomophthorales) strain number 3491, originally isolated from *Dendrolaelaps* spp. by Professor Stanisław Bałazy (Polish Academy of Science, Research Center for Agricultural and Forest Environment, Poznań), was used for infection. The isolate used for research is stored at the Museum and Institute of Zoology Polish Academy of Sciences. To increase virulence, fungal colonies were grown on Sabouraud agar medium supplemented with homogenized *G. mellonella* larvae to a final concentration of 10% (SAB-GM). To stimulate sporulation, the fungus was incubated at 20 °C under a 12 h photoperiod (L:D 12:12) [69]. The fungal SAB-GM colonies were cultured for seven days. These were used for all experiments.

Conidia were harvested from sporulating SAB-GM colonies by rinsing them with sterile water. The collected conidia were divided into three groups, each of which was treated differently: part one (P1) was stained with Calcofluor White (Merck, Darmstadt, Germany), as described previously [45]; part two (P2) was incubated in a Petri dish with sterile water at room temperature for 24 h to enable germination; and part three (P3) was immediately frozen at −20 °C to prevent germination.

The number of conidia in each collected sample was counted in a Bürker chamber using light microscopy, as described previously [45]. The microscope observations and photo documentation of conidia, hyphae and insect cuticles were performed using an Axio Vert.A1 fluorescence microscope (Zeiss, Jena, Germany) with Axio Cam 305 color (Zeiss, Jena, Germany) and the ZEN 3.2 lite software with Modul Image Analysis (Zeiss, Jena, Germany).

### 2.3. Fungal Infection

The tested insects were subjected to 24 h contact with sporulating fungal colonies at 20 °C. Around 20 larvae of *G. mellonella* or *C. vicina* were placed in each 90 mm Petri dish containing a *C. coronatus* colony. The controls consisted of larvae exposed to sterile SAB-GM medium. This method of triggering insect infection by *C. coronatus* in laboratory conditions is considered the most effective and closest to the natural infection process [11]. Adult insects (approximately 30 flies or moths) were placed for 24 h in a plastic container with a Petri dish without a lid containing either a fungal colony or sterile SAB-GM. After the exposure, the insects were either immediately frozen at −20 °C or transferred to clean containers with food and placed for 24 h in the optimal growth conditions described in Section 2.1.

The insects that had contact with the fungus were rinsed with 1 mL of sterile water to collect any spores. The washed-out spores were transferred to microcentrifuge tubes (1.5 mL) and frozen to stop spore germination before being counted: the aim was to determine the number of spores that accumulated on the cuticles of insects exposed to the fungal colony, thus establishing the rate of recovery of the spores present on the cuticles. Briefly, 20 µL of sterile water containing 1500 spores was applied to the backs of the larval and adult *G. mellonella* and *C. vicina*. After 30 min of incubation, the spores were rinsed off from the insects with 1 mL of sterile water and immediately frozen.

To visualize the fungal spores and hyphae on the surfaces of insects following contact with the fungus, the larvae and adults were stained with Calcofluor White and observed under an Axio Vert.A1 fluorescence microscope (Zeiss, Jena, Germany), as described above.

### 2.4. Extraction of Samples, Derivatization and GC/MS Analysis

To isolate the surface lipid components for GC/MS analysis, the frozen insects were extracted for five minutes in 20 mL of dichloromethane (Merck, Darmstadt, Germany). The extracts were then placed in glass flasks and evaporated under nitrogen as described earlier [11]. The same extraction procedure was applied to *C. coronatus* conidia.

The extracts were treated with 100 μL of BSTFA:TMCS mixture 99:1 (Merck, Darmstadt, Germany) and heated for one hour at 100 °C to form trimethylsilyl esters (TMSs). The TMSs of the fatty acids were then analyzed via GC/MS using a GCMS-QP2010 system with a mass detector (Shimadzu, Kioto, Japan) and the NIST 11 library. The analysis used 19-methylarachidic acid (Merck, Darmstadt, Germany; 1 mg/mL) as an internal standard (IS) because it separates well from all the sample constituents and was not previously detected in the insect samples [11].

The mass spectra of the tested trimethylsilyl esters revealed the presence of an M+ (molecular ion), [M-15]+, and fragment ions at *m*/*z* 117, 129, 132. The contents were calculated by comparing the relative peak areas with the IS peak area. All constituents were assumed to have response factors of one. Helium was used as the carrier gas. The analysis was performed in split injection mode using a ZB-5MSi (Zebron, Phenomenex, Torrance, CA, USA) column (thickness, 0.25 µm; length, 60 m; diameter, 0.25 µm). The column oven temperature cycle was held at 80 °C for three minutes and then ramped from 80 °C to 310 °C at 4 °C/min; the final temperature was then held for 10 min. The ion source temperature was 200 °C; the interface temperature was 310 °C. This method is described in more detail elsewhere [11,46,47,66,67].

### 2.5. Statistics

Student’s *t*-test and ANOVA were used to check the statistical significance of the results. Normality was checked using the Kolmogorov–Smirnov (K-S) test. Post hoc analysis was performed using the Tukey test. Results at the *p* ≤ 0.05 level were considered significant. The analysis was performed using the STATISTICA 6.0 (StatSoft Polska, Kraków, Poland) and Prism 8.0 software (GraphPad Software, Boston, MA, USA).

## 3. Results

### 3.1. Effect of Fungal Infection on Insects

**Figure 1:**
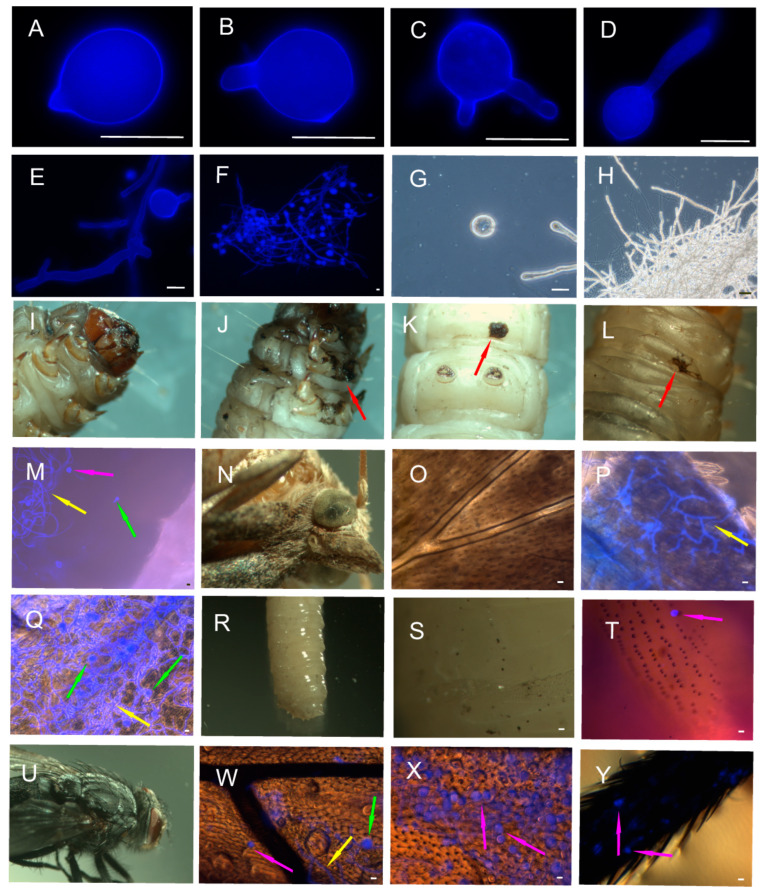
Impact of *Conidiobolus coronatus* infective structures on insect cuticles. *C. coronatus* conidia and hyphae (**A**–**H**): stained with calcofluor (**A**–**F**), unstained observed in phase contrast (**G**,**H**), germinating conidia (**B**–**E**). *Galleria mellonella* larvae (**I**–**M**) and adults (**N**–**Q**) treated with *C. coronatus*: larvae (**J**–**M**), adults (**P**,**Q**). *G. mellonella* controls (exposed to sterile SAB-GM): larva (**I**), adult’s head (**N**), adult’s wing (**O**). *Calliphora vicina* larvae (**R**–**T**) and adults (**U**–**Y**) treated with *C. coronatus*: larvae (**S**–**T**), adults (**W**–**Y**). *C. vicina* controls (exposed to sterile SAB-GM): larva (**R**), adult (**U**). Insects treated with fungus and stained with calcofluor: *G. mellonella* larva (**M**), *G. mellonella* adult wing (**P**), *G. mellonella* adult thorax (**Q**), *C. vicina* larva (**T**), *C. vicina* adult wing (**W**), *C. vicina* adult thorax (**X**), *C. vicina* adult leg (**Y**). Red arrows: cuticle penetration site; pink arrows: conidia attached to the cuticle; green arrows: conidia germinating on the cuticle; yellow arrows: hyphae. Scale bars, 20 µm.

The effect of contact with *C. coronatus* sporulating colonies was fully consistent with what we observed in our previous studies: high mortality (90%) of *G. mellonella* larvae, low mortality (10%) of adult moths, total resistance of *C. vicina* larvae (mortality 0%) and high mortality of adult flies (95%) resulting from their licking the surface of the fungal colonies. Following infection, dark, melanized spots appeared on the cuticles of the infected *G. mellonella* larvae (Figure 1J–L); the insects stopped spinning cocoons and became immobile [15,46].

Unfortunately, the recovery rates of the *C. coronatus* spores applied to the insect cuticles were very low (4–8%), only reaching 33% in the case of wax moth larvae (Table 1). This made it impossible to estimate how many spores accumulated on the cuticles of the insects during their exposure to the sporulating colonies.

Staining with Calcofluor White (Figure 1) showed the presence of numerous germinating spores and hyphae on the cuticles of *G. mellonella* larvae, and a large number of these structures were on the wings and thoraxes of adult moths. In contrast, the few spores present on the cuticles of *C. vicina* larvae did not germinate. Contact between adult flies and the sporulating fungus also resulted in the appearance of hyphae from germinating spores on the wings and thoraxes but far fewer than found in *G. mellonella*. Spores were also found on the legs of adult flies, but none germinated.

Almost all spores (97% on average) germinated when left in water for 24 h. In these samples, primary spores dominated, while secondary spores were rarely observed (less than 2%). The presence of microconidia and hairy spores was not observed in any of the tested samples.

### 3.2. The Influence of C. coronatus on the Cuticular FFA Profiles of Galleria mellonella

GC/MS analysis indicated both quantitative and qualitative differences in the FFA content of lipids extracted from the *G. mellonella* cuticle between the control larvae and adults, as well as between the controls and infected insects (Table 2). The total cuticular FFA content was 485.49 µg/g of body mass in the control larvae and 6417.28 µg/g of body mass in the control adults (13.2 times higher). In addition, 15 compounds from C4:0 to C20:1 were identified in the control larvae, and 16 from C6:0 to C20:1 were identified in the control adults. In addition, C4:0, C5:0 and C7:0 were present in the control larvae but absent in the adult controls, while C11:0, C12:0, C13:0 and C18:0 were absent in the larvae but present in the adults. In the case of FFAs occurring in the cuticle extracts of both larvae and adults, the concentrations ranged from 1.5 (C14:0) to 83.6 (C9:0) times higher in the adults. The highest concentrations in the control moths were observed for C16:0 (3687.79 µg/g of body mass) and C11:0 (1268.38 µg/g of body mass; C11:0 was absent in larvae).

The FFA profiles of *G. mellonella* were significantly affected by *C. coronatus* infection. In the larvae, the total cuticular FFA content increased immediately following exposure to the fungus, reaching 6241.45 µg/g body mass (12.8 times higher) 24 h post-infection (hpi); although it then fell to 2063.45 µg/g body mass after another 24 h (48 hpi, dying larvae), this level was still 4.2 times higher than in the control larvae (Table 2). In contrast, among the adults, exposure resulted in a decrease in total cuticular FFA content to 2055.45 µg/g body mass in the 24 hpi group (3.1 times lower) and 101.58 µg/g body mass in the 48 hpi group (63.2 times lower).

Contact with the fungus resulted in the disappearance of C4:0, C5:0, C14:1 and C20:1 from the larval cuticle, accompanied by the appearance of C12:0 (in the 24 hpi group) and C13:0 (in both the 24 and 48 hpi groups); in addition, the concentrations of C14:0, C15:0, C16:1, C16:0 and C18:1 increased. Among adults, exposure resulted in the appearance of C7:0 (not present in unexposed control moths) and the disappearance of C14:1, C16:1 (only in the 24 hpi group), C18:2 and C20:1. In addition, the concentration of C9:0, C11:0, C16:0, C18:1 and C18:0 significantly fell in the 24 hpi and 48 hpi groups; however, C13:0 and C14:0 were higher than the control values at 24 hpi but significantly lower at 48 hpi.

### 3.3. The Influence of C. coronatus Treatment on the Cuticular FFA Profiles of Calliphora vicina

Extracts of cuticular lipids obtained from *C. vicina* demonstrated significantly lower FFA content than those from *G. mellonella* (larvae, 10.8 times lower; adults, 72.8 times lower; Table 2 and Table 3). Approximately twice the FFA level was extracted from the control adults (88.13 µg/g of body mass) than the control larvae (44.82 µg/g). Eight FFAs from C12:0 to C18:0 were detected in the control larvae, while seventeen FFAs from C6:0 to C24:0 were identified in the control adult flies, of which C6:0, C8:0, C9:0, C10:0, C15:0, C17:0, C18:2, C20:0, C22:0 and C24:0 were absent from the control larvae. In addition, C12:0 was present in the larvae but absent in the control adults. No differences in the concentrations of C14:1 or C16:1 were found between the larvae and flies, while C14:0, C16:0, C18:1 and C18:0 concentrations were 1.3 to 4.5 times higher in adults (Table 3).

Although the exposure of the *C. vicina* larvae to the fungus did not result in mycosis, it nevertheless exerted a profound effect on the cuticular FFA profile, resulting in the appearance of C13:0 and C15:0 (both absent in the control larvae) and the disappearance of C17:1; it also increased the levels of C12:0, C14:0, C16:1, C16:0, C18:1 and C18:0 by between 3.1 and 1009.3 times compared with the control values. The accumulation of these FFAs in the larval cuticles was a progressive and dynamic process, and in each case, the values in the 48 hpi group were much higher than in the 24 hpi group. Hence, the total FFA contents in the 24 and 48 hpi groups were, respectively, 26.4 (1182.35 µg/g of body mass) and 94.6 (4239.99 µg/g of body mass) times higher than in control larvae (44.82 µg/g of body mass) (Table 3).

Among the adult flies, contact with fungal colonies resulted in death two to three days later; infection most probably occurred because of the fly licking the mycelium covered with conidia. After infection, their cuticular FFA profiles were characterized by the disappearance of C14:1, C17:1, C17:0, C18:2, C20:0, C22:0 and C24:0 and the appearance of C12:0 (in the 24 hpi group only) and C13:0 (in both the 24 and 48 hpi groups). Fungal infection also increased the concentration of C6:0, C8:0 and C9:0 24 h after contact with the fungus, followed by the complete disappearance of these FFAs after another 24 h; however, in dying flies (48 hpi), the elevated levels of C14:0, C15:0, C16:1, C16:0, C18:1 and C18:0 increased even further, ranging from 12.3 to 137 times higher than in the control group. The total FFA contents in the 24 and 48 hpi groups were, respectively, 2.3 (205.02 µg/g of body mass) and 17 (1498.75 µg/g of body mass) times higher than in the control adults (88.13 µg/g of body mass) (Table 3).

### 3.4. FFA Content in Conidiobolus coronatus Conidia

The lipids extracted from the conidia were then analyzed to estimate the share of FFAs present in the *C. coronatus* spores attached to the cuticles of insects in the total FFA profiles of the exposed insects. Given that *C. coronatus* spores germinated on the *G. mellonella* cuticles but hardly ever on the *C. vicina* cuticles (Figure 1), the FFA profiles of the non-germinated spores (freshly harvested from the fungus colony) were compared with those of the germinated spores, i.e., following 24 h incubation in sterile water.

**Figure 2:**
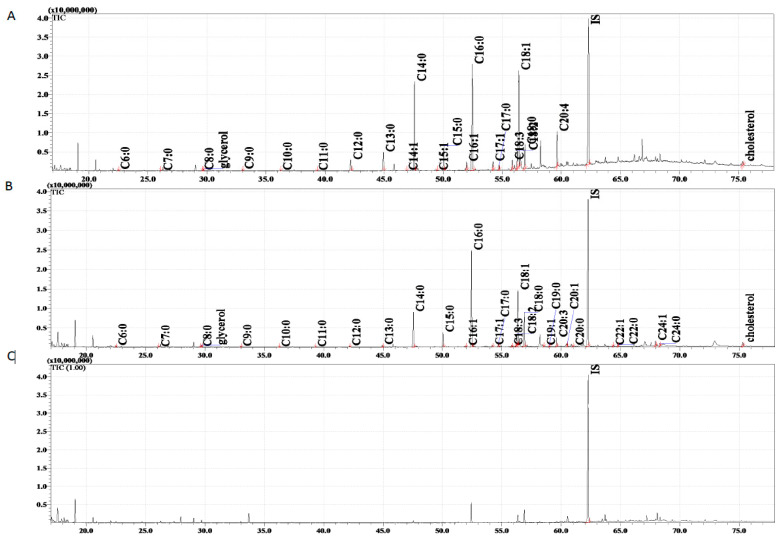
The total ion current (TIC) of fatty acids (TMS esters) of the dichloromethane extract from *Conidiobolus coronatus* conidia: (**A**) non-germinated, (**B**) germinated, (**C**) sterile water. IS—internal standard 19-methylarachidic acid; fatty acids and molecular ions: hexanoic acid (C6:0, *m*/*z* = 188), heptanoic acid (C7:0, *m*/*z* = 202), octanoic acid (C8:0, *m*/*z* = 216), nonanoic acid (C9:0, *m*/*z* = 230), decanoic acid (C10:0, *m*/*z* = 244), undecanoic acid (C11:0, *m*/*z* = 258), dodecanoic acid (C12:0, *m*/*z* = 272), tridecanoic acid (C13:0, *m*/*z* = 286), tetradecenoic acid (C14:1, *m*/*z* = 298), tetradecanoic acid (C14:0, *m*/*z* = 300), pentadecenoic acid (C15:1, *m*/*z* = 312), pentadecanoic acid (C15:0, *m*/*z* = 314), hexadecenoic acid (C16:1, *m*/*z* = 326), hexadecanoic acid (C16:0, *m*/*z* = 328), heptadecenoic acid (C17:1, *m*/*z* = 340), heptadecanoic acid (C17:0, *m*/*z* = 342), octadecatrienoic acid (C18:3, *m*/*z* = 350), octadecadienic acid (C18:2, *m*/*z* = 352), octadecenoic acid (C18:1, *m*/*z* = 354), octadecanoic acid (C18:0, *m*/*z* = 356), nonadecenoic acid (C19:1, *m*/*z* = 368), nonadecanoic acid (C19:0, *m*/*z* = 370), eicozenoic acid (C20:1, *m*/*z* = 367), eicosatetraenoic acid (C20:4, *m*/*z* = 376), docosenoic acid (C22:1, *m*/*z* = 410), docosanoic acid (C22:0, *m*/*z* = 412), etracosenoic acid (C24:1, *m*/*z* = 438), etracosanoic acid (C24:0, *m*/*z* = 440).

GC/MS analysis (Figure 2) identified 21 FFAs from C6:0 to C20:4 in non-germinated spores and 27 FFAs in germinated spores (Table 4). Germination was accompanied by the disappearance of C14:1, C15:1 and C20:4 and the appearance of C19:1, C19:0, C20:3, C20:1, C20:0, C22:1, C22:0, C24:1 and C24:0, all of which were absent from the non-geminated conidia.

In both experimental groups, the concentrations of individual FFAs in the germinated conidia were 1.23 to 19.89 times lower than in the non-germinated ones, and the total FFA concentration was 3.25 times lower (Table 4). GC/MS analyses also indicated the presence of glycerol and cholesterol, whose respective concentrations significantly decreased after germination from 198 ± 5 to 129 ± 2 fg/conidium (1.53 times, *p* < 0.0001) and from 558 ± 7 to 387 ± 81 fg/conidium (1.44 times, *p* < 0.05).

## 4. Discussion

Insects have evolved two main defense systems protecting them against pathogens: the cuticle, which serves as the primary barrier, and the immune system. The main purpose of the cuticle is to protect against the intrusion of pathogens into the body cavity. When this protection fails, the cellular and humoral immune systems take up the fight. The extremely diverse structure of the cuticles of various insect species and the varied efficiency of their immune systems are reflected in their varied susceptibility to fungal infection [15,24,25,26,27,61,70,71,72,73,74].

As a typical representative of the Entomophthorales, *C. coronatus* has a highly selective action on insects: susceptible insect species are killed quickly and efficiently, while resistant species remain unhurt [5,13,15]. When *C. coronatus* spores attach to the cuticle of a susceptible insect species, they germinate rapidly and form invasive hyphae that secrete enzymes hydrolyzing cuticular proteins, chitin and lipids, thus breaking the integrity of the cuticle and allowing the fungus to enter and colonize the body cavity [14,15,63]. However, if spores land on the cuticle of a resistant species, they will not germinate. This is the case of fly larvae [15] and partially adult flies: a few conidia germinated on *C. vicina* wings, but none did so on adult thoraxes or legs (Figure 1). The high mortality demonstrated by adult scavenger flies following contact with *C. coronatus* colonies has been attributed to their behavior, as the flies lick all substrates including the surfaces of fungal colonies, thus providing access to the digestive tract for the spores and toxic secondary metabolites of the fungus [73].

Our recent studies confirmed the belief that the chemical composition of the cuticle is the main factor determining susceptibility or resistance to fungal infection [15,44,45,46,47]. The crucial aspect seems to be the composition of the epicuticle, the outermost cuticle surface, which comprises a mixture of lipids, proteins and phenolic compounds covered by a layer of hydrocarbons, fatty acids, esters, alcohols, sterols and aldehydes [24,25,61,63,74]. Several cuticular fatty acids and their methyl esters and alcohols, as well as various atypical compounds detected on the cuticles of *C. coronatus*-resistant insect species, are presently known to inhibit its growth, sporulation, virulence and toxicity and to influence the activity of fungal enzymes that degrade insect cuticles (CDEs) [44,45,46,47]. However, several other compounds present on the insect cuticle also enhance fungus sporulation, virulence and CDE activity, indicating that the compensation strategies employed by *C. coronatus* are very complex and that it has high plasticity [44,45]. One significant example is that adding C16 fatty alcohol to an *in vitro* culture of *C. coronatus* greatly reduces sporulation while strongly increasing the activity of CDEs, thus maintaining the high virulence of the fungus [45].

Understanding the role played by cuticular components in the defense of insects against fungal attack is crucial for the development of new methods of using fungi and/or their secondary metabolites as natural insecticides. In turn, identifying compounds that protect insects against mycosis may be essential for developing strategies for preventing mycoses in humans and farm animals. In this sense, *C. coronatus* is a very promising research model: conidiobolomycosis has often been recorded in humans, horses and sheep in tropical regions, as well as in insects [75,76,77], and given the close similarity between their innate immune mechanisms, it appears that mammals can be replaced with insect models. This would not only reduce costs but also avoid the ethical problems associated with using mammals in research [78]. The current work examines how contact with *C. coronatus* affects the lipid profile of the cuticles of susceptible insects (*G. mellonella*) and compares it with that of resistant insects (*C. vicina*).

In *G. mellonella* larvae, contact with the fungus resulted in the appearance of C12:0 and C13:0, which were not present in the controls, and an increase in C14:0, C15:0, C16:1, C16:0, C18:1 and C18:0. In contrast, in the adults, the same treatment resulted in the appearance of C7:0 and the elevation of C13:0, C14:0 and C15:0 levels. Both larvae and adults accumulated C13:0, C14:0 and C15:0 after contact with the fungus, suggesting the activation of common synthesis mechanisms and the active transport of these FFAs to the cuticle; however, the precise nature of these processes is currently unknown. Exposure to the fungus also resulted in the disappearance of several FFAs (C4:0, C5:0, C14:1 and C20:1 in larvae; C14:1, C18:2 and C20:1 in adults) and a reduction in the levels of C6:0, C9:0, C11:0, C16:0, C18:1 and C18:0 in the adults. This also indicates the presence of significant physiological differences between larvae and adults and may reflect the course of the fatal disease processes in larvae and the deterioration of the condition in adults, despite the lack of external signs.

Similar changes in FFA profiles were observed in *C. vicina* larvae and adults exposed to the fungus. In the larvae, contact with *C. coronatus* resulted in the appearance of C11:0 (not present in the controls) and an increase in C12:0, C16:1, C16:0, C18:1 and C18:0 levels; in the adults, the treatment resulted in the appearance of C12:0 and C13:0 (absent in control flies) and an increase of C14:0, C15:0, C16:1, C16:0, C18:1 and C18:0. Similar effects were also observed when adult *Sarcophaga argyrostoma* (Diptera: Sarcophagidae) flies were exposed to *C. coronatus* [73].

It can be seen that despite being systematically distant, leading different lifestyles and living in different environments, contact with the fungus elicited similar responses in FFA content in both species; this suggests that FFAs play a role in the defense against this pathogen. Indeed, *in vitro* studies have found that the virulence of *C. coronatus* is inhibited by adding C12:0, C14:0, C15:0, C16:0 or C18:1 to the culture medium; in addition, C16:1 reduces the toxicity of post-incubation filtrates and biomass production but stimulates sporulation. In turn, C16:0, C18:0 and C18:1 all reduce the production of biomass and spores [44]. It is not known whether C13:0 has any impact on the pathogenicity of *C. coronatus* as it has not yet been tested.

The disappearance of C17:1, C17:0, C18:2, C20:0, C22:0 and C24:0 from the cuticles of adult flies following contact with *C. coronatus* is probably connected to their deaths due to the consumption of fungal spores and/or toxic excretions. In both insect species, contact between the larvae and fungus stimulated the selective accumulation of FFAs in cuticles, reflected in a significant increase in total FFA content; however, while the adult *C. vicina* accumulated FFAs, albeit to a lesser extent than the larvae, the adult *G. mellonella* lost their cuticular FFAs. The mechanisms governing the processes leading to FFA accumulation remain obscure, but it is obvious that different defense strategies are used by the two species [15].

It is possible that the lipid profile of the fungal spores may influence that of the cuticle following exposure: after settling on the cuticle, the spores either germinated and formed hyphae, i.e., in the case of *G. mellonella* larvae and adults and to a smaller extent in the case of *C. vicina* wings, or did not germinate, i.e., in *C. vicina* larvae and the legs and thoraxes of adult flies. Hence, the lipids from non-germinating and germinating spores were subjected to chromatographic analysis. However, although we originally intended to present the quantitative results in terms of the number of settled spores, this was not possible because of the extremely low recovery rates (Table 1). Among adult moths, the low recovery rate may be due to the rapid germination of the spores and their binding to the wing scales; however, it is much more difficult to explain the low recovery rate for fly larvae, with an almost smooth surface with few hairs, and adult flies, in which only single spores germinated on the membranous wings and none germinated on the heavily hairy thoraxes or legs. These observations are in agreement with previous data concerning the effectiveness of cuticle degradation caused by *C. coronatus* CDEs: in *C. vicina* adults, both the proteolytic and chitinolytic enzymes demonstrated lower hydrolysis efficiency on the cuticle of the thorax compared with that of the wings [63]. This indicates that, across the body of a fly, significant dissimilarities exist in the spatial distribution of the cuticular components that efficiently prevent the germination of *C. coronatus* spores. The graded properties of frequently patterned insect cuticles produced through changes in material composition, density and microstructure are likely to facilitate their function as protective barriers against pathogens and mechanical stresses [79,80,81].

As it was not possible to accurately determine the number of spores that accumulated on the cuticles after exposure, this study focused on the qualitative contribution of individual spore fatty acids to the overall FFA profile of the infected insects. However, this task was complicated by the fact that the same fatty acids occur both in the tested insects and in the spores. No firm conclusions can hence be drawn from the obtained data. In *G. mellonella*, C12:0 and C13:0 were absent from the cuticles of the control larvae but present in very high concentrations in the infected larvae (Table 2). It is possible that their presence in the cuticles of the infected larvae could result from the attached fungal spores, but no such relationship was confirmed in the adult moths, where both acids were detected in the controls. In addition, the massive presence of germinated conidia (Figure 1) had no effect on the C12:0 level. However, both acids were also present at much higher levels in *C. vicina* larvae and adults treated with the fungus (Table 3). It is currently difficult to determine whether the large increases observed in C14:0 and C16:0 concentrations in the cuticular extracts were due to the high concentrations present in the conidia or to the activation of the insect defense response. Resolving this problem will require further research.

A detailed discussion of the qualitative and quantitative changes in the lipid profile of germinating spores, although undoubtedly interesting for researchers of the physiological processes of fungi, goes beyond the scope of *Insects*.

## Figures and Tables

**Table 1:** Recovery rates (% ± SD of the mean) of *C. coronatus* spores settled on the insect cuticles.

Insect Species	Larvae	Adults
*Galleria mellonella*	33 ± 11	8 ± 11
*Calliphora vicina*	5 ± 7	4 ± 6

In total, 1500 spores of *C. coronatus* suspended in 20 µL of sterile water were applied to the backs of larvae and adults. After 30 min of incubation on the cuticles, the spores were rinsed with 1 mL of sterile water and counted as described in Section 2. Each variant was performed in triplicate. Differences were statistically insignificant (*t*-test, ANOVA). SD—standard deviation.

**Table 2:** Free fatty acid content present on the cuticles of *Galleria mellonella* larvae and adults (µg/g of body mass ± SD).

	*Galleria mellonella* Larvae (µg/g of Body Mass ± SD)			*Galleria mellonella* Adults (µg/g of Body Mass ± SD)		
FFA	Control	Fungal Infection 24 hpi	Fungal Infection 48 hpi	Control	Fungal Infection 24 hpi	Fungal Infection 48 hpi
C_4:0_	0.72 ± 0.28	ND	ND	ND	ND	ND
C_5:0_	0.36 ± 0.13	ND	ND	ND	ND	ND
C_6:0_	1.11 ± 0.47	1.95 ± 0.69	1.29 ± 0.65	2.04 ± 1.03	0.67 ± 0.25	0.03 ± 0
C_7:0_	0.96 ± 0.34	0.90 ± 0.92	0.68 ± 0.23	ND	0.60 ± 0.57	0.02 ± 0
C_8:0_	1.63 ± 0.05 ^A^	2.93 ± 1.68	1.80 ± 0.35	4.36 ± 1.47 ^A,l^	2.60 ± 0.69 ^m^	0.27 ± 0.01 ^l,m^
C_9:0_	1.34 ± 0.17 ^B^	3.36 ± 1.39 ^a^	3.86 ± 0.85 ^a^	112.02 ± 6.48 ^B,n^	44.36 ± 1.17 ^n^	4.82 ± 0.20 ^n^
C_10:0_	0.96 ± 0.30 ^b^	3.04 ± 0.98 ^b,c^	0.75 ± 0.17 ^c^	4.37 ± 3.18	3.31 ± 1.21 ^o^	0.17 ± 0 ^o^
C_11:0_	ND	ND	ND	1268.38 ± 361.77 ^p^	424.06 ± 10.48 ^p^	29.71 ± 0.67 ^p^
C_12:0_	ND	214.66 ± 24.19	ND	23.94 ± 5.77 ^q^	20.87 ± 1.70 ^r^	0.32 ± 0.01 ^q,r^
C_13:0_	ND	121.50 ± 13.35 ^d^	12.84 ± 1.59 ^d^	7.92 ± 0.25 ^s^	52.19 ± 2.30 ^s^	0.64 ± 0.03 ^s^
C_14:1_	2.02 ± 1.35	ND	ND	12.12 ± 6.61	ND	ND
C_14:0_	28.69 ± 9.39 ^e^	519.57 ± 41.47 ^e^	71.73 ± 9.65 ^e^	43.94 ± 13.15 ^t^	209.09 ± 4.93 ^t^	1.28 ± 0.07 ^t^
C_15:0_	7.12 ± 5.79 ^f^	151.04 ± 13.18 ^f,g^	14.68 ± 7.35 ^g^	17.93 ± 11.24 ^u^	55.01 ± 2.19 ^u^	0.36 ± 0 ^u^
C_16:1_	18.34 ± 13.72 ^C,h^	90.37 ± 14.67 ^h^	41.63 ± 3.67 ^h^	264.61 ± 9.36 ^C,v^	ND	1.34 ± 0.03 ^v^
C_16:0_	329.52 ± 154.13 ^D,i^	4680.44 ± 273.90 ^i^	1676.10 ± 246.23 ^i^	3687.79 ± 323.09 ^D,w^	905.38 ± 14.58 ^w^	51.54 ± 2.02 ^w^
C_18:2_	ND	ND	ND	62.52 ± 84.21	ND	ND
C_18:1_	80.19 ± 49.57 ^E,j^	410.94 ± 21.45 ^j^	238.09 ± 38.94 ^j^	730.16 ± 142.87 ^E,x^	315.02 ± 17.77 ^x^	9.92 ± 0.07 ^x^
C_18:0_	12.27 ± 2.72 ^F^	40.74 ± 24.93	ND	168.87 ± 10.78 ^F,y^	22.27 ± 6.44 ^y^	1.14 ± 0 ^y^
C_20:1_	1.33 ± 0.37 ^G^	ND	ND	6.29 ± 2.83 ^G^	ND	ND
Sum of FFAs	485.49 ± 238.78 ^H,k^	6241.45 ± 432.83 ^k^	2063.45 ± 309.69 ^k^	6417.28 ± 984.12 ^H,z^	2055.45 ± 64.33 ^z^	101.58 ± 3.13 ^z^

Statistically significant differences (*t*-test, ANOVA, Tukey’s HSD Test, *p* < 0.05) are marked with the same letters. Capital letters (A–H) indicate significant differences between control larvae and adults. Small letters indicate significant differences between control and infected larvae (a–k) and adults (l–z). SD—standard deviation; FFA—free fatty acid; ND—not detected; hpi—hours post-infection.

**Table 3:** Free fatty acid content present on the cuticles of *Calliphora vicina* larvae and adults (µg/g of body mass ± SD).

	*Calliphora vicina* Larvae (µg/g of Body Mass ± SD)			*Calliphora vicina* Adults (µg/g of Body Mass ± SD)		
FFA	Control	Fungal Infection 24 hpi	Fungal Infection 48 hpi	Control	Fungal Infection 24 hpi	Fungal Infection 48 hpi
C_6:0_	ND	ND	ND	0.09 ± 0.02 ^i^	0.51 ± 0.11 ^i^	ND
C_8:0_	ND	ND	ND	0.12 ± 0.01 ^j^	1.19 ± 0.83 ^j^	ND
C_9:0_	ND	ND	6.28 ± 1.41	0.45 ± 0.02 ^k^	1.24 ± 0.03 ^k^	ND
C_10:0_	ND	ND	3.29 ± 0.59	0.13 ± 0.02	ND	ND
C_12:0_	0.25 ± 0.13 ^a^	24.48 ± 1.12 ^a^	117.65 ± 36.11 ^a^	ND	2.29 ± 0.11	ND
C_13:0_	ND	54.57 ± 1.23 ^b^	245.09 ± 71.08 ^b^	ND	2.83 ± 0.09 ^l^	20.46 ± 11.01 ^l^
C_14:1_	0.49 ± 0.23	ND	ND	0.39 ± 0.06	ND	ND
C_14:0_	1.06 ± 0.48 ^c^	215.08 ± 6.73 ^c^	1069.82 ± 302.58 ^c^	1.41 ± 0.06 ^m^	18.01 ± 1.08 ^m^	193.11 ± 39.11 ^m^
C_15:0_	ND	60.41 ± 3.28	175.54 ± 113.09	0.49 ± 0.01 ^n^	3.46 ± 0.24 ^n^	28.12 ± 4.97 ^n^
C_16:1_	24.46 ± 3.19 ^d^	76.69 ± 3.92 ^d^	150.13 ± 54.48 ^d^	23.84 ± 1.57 ^o^	17.89 ± 0.91 ^o^	295.08 ± 67.26 ^o^
C_16:0_	9.73 ± 0.82 ^A,e^	609.55 ± 26.15 ^e^	2053.95 ± 661.04 ^e^	21.81 ± 0.85 ^A,p^	72.08 ± 2.99 ^p^	519.80 ± 100.70 ^p^
C_17:1_	0.55 ± 0.08 ^B^	ND	ND	0.19 ± 0.02 ^B^	ND	ND
C_17:0_	ND	ND	ND	0.77 ± 0.12	ND	ND
C_18:2_	ND	ND	ND	2.19 ± 0.05	ND	ND
C_18:1_	6.04 ± 0.49 ^C,f^	120.37 ± 2.73 ^f^	315.81 ± 34.52 ^f^	26.98 ± 1.61 ^C,q^	61.70 ± 1.69 ^q^	344.02 ± 107.75 ^q^
C_18:0_	2.23 ± 0.27 ^D,g^	21.20 ± 0.95 ^g^	102.40 ± 20.68 ^g^	3.82 ± 0.05 ^D,r^	23.81 ± 1.32 ^r^	98.15 ± 22.49 ^r^
C_20:0_	ND	ND	ND	0.85 ± 0.55	ND	ND
C_22:0_	ND	ND	ND	2.88 ± 0.04	ND	ND
C_24:0_	ND	ND	ND	1.71 ± 0.19	ND	ND
C_26:0_	ND	ND	ND	ND	ND	ND
Sum of FFAs	44.82 ± 5.70 ^E,h^	1182.35 ± 46.12 ^h^	4239.99 ± 1295.59 ^h^	88.13 ± 5.29 ^E,s^	205.02 ± 9.44 ^s^	1498.75 ± 353.32 ^s^

Statistically significant differences (*t*-test, ANOVA, Tukey’s HSD Test, *p* < 0.05) are marked with the same letters. Capital letters (A–E) indicate significant differences between control larvae and adults. Small letters indicate significant differences between control and infected larvae (a–h) and adults (i–s). SD—standard deviation; FFA—free fatty acid; ND—not detected; hpi—hours post-infection.

**Table 4:** Free fatty acid content in *Conidiobolus coronatus* conidia (femtogram/conidium ± SD).

	FFA Concentration (fg/Conidium ± SD)	
FFA	Non-Germinated Spores	Germinated Spores
C6:0	149 ± 7 ^a^	83 ± 8 ^a^
C7:0	21 ± 2	17 ± 10
C8:0	70 ± 5 ^b^	27 ± 1 ^b^
C9:0	188 ± 2 ^c^	49 ± 2 ^c^
C10:0	53 ± 3 ^d^	9 ± 1 ^d^
C11:0	33 ± 3 ^e^	6 ± 2 ^e^
C12:0	1631 ± 71 ^f^	82 ± 6 ^f^
C13:0	2695 ± 97 ^g^	169 ± 2 ^g^
C14:1	47 ± 6	ND
C14:0	14,735 ± 242 ^h^	2490 ± 62 ^h^
C15:1	20 ± 6	ND
C15:0	3507 ± 103 ^i^	1063 ± 51 ^i^
C16:1	907 ± 36 ^j^	76 ± 6 ^j^
C16:0	15,797 ± 172 ^k^	6928 ± 105 ^k^
C17:1	1000 ± 44 ^l^	138 ± 8 ^l^
C17:0	550 ± 38 ^m^	223 ± 10 ^m^
C18:3	1112 ± 98 ^n^	90 ± 8 ^n^
C18:2	2203 ± 144 ^o^	767 ± 148 ^o^
C18:1	12,346 ± 439 ^p^	3571 ± 289 ^p^
C18:0	2692 ± 108 ^q^	1913 ± 82 ^q^
C19:1	ND	53 ± 8
C19:0	ND	28 ± 1
C20:4	4068 ± 123	ND
C20:3	ND	531 ± 4
C20:1	ND	236 ± 83
C20:0	ND	81 ± 12
C22:1	ND	77 ± 32
C22:0	ND	130 ± 25
C24:1	ND	485 ± 182
C24:0	ND	267 ± 84
Sum of FFAs	63,827 ± 1643 ^r^	19,588 ± 1015 ^r^

Statistically significant differences (*t*-test, *p* < 0.05) are marked with the same letters. SD—standard deviation; FFA—free fatty acid; ND—not detected.

## Data Availability

The data presented in this study are available upon request from the corresponding authors. The data are not publicly available because of ongoing renovation works related to the planned move of the Museum and Institute of Zoology of the Polish Academy of Sciences to a new location. Work on creating the data repository has not yet been completed.
